# Giant Polycystic Papillary Renal Cell Carcinoma: A Case Report and Literature Review

**DOI:** 10.3389/fonc.2022.876217

**Published:** 2022-05-12

**Authors:** Zhongming Huang, Hai Wang, Zhigang Ji

**Affiliations:** Department of Urology, Peking Union Medical College Hospital, Chinese Academy of Medical Science and Peking Union Medical College, Beijing, China

**Keywords:** papillary renal cell carcinoma, laparoscopic surgery, PET-CT, Bosniak classification, pathology

## Abstract

**Introduction:**

Giant, cystic renal tumors are generally considered relatively contraindicated for laparoscopic surgery. We report on a 19-year-old male, where polycystic lesions in the left kidney were accidentally noted by enhanced computed tomography (CT) by focusing on the diagnostic, clinical, and surgery to the patient.

**Case Report:**

Enhanced CT scan revealed solid component in multiple cystic lesions of Bosniak IV, which was enhanced after injection of contrast agent and the left kidney lost normal profile and enlarged with maximal diameter more than 18cm. Positron emission tomography-computed tomography (PET-CT) showed SUVmax 4.8 of the lesion and suggested malignant disease. A retroperitoneal laparoscopic radical left nephrectomy was performed successfully without cyst burst and the lesion was 17×17×18 cm in size. Pathological examination revealed that the lesions were consistent with papillary renal cell carcinoma (type 2, WHO grade II), no renal capsule invasion, no renal pelvis and renal sinus fat involvement, no abnormality in ureter and renal arteriovenous end, no abnormality in a few adrenal tissues, chronic inflammation of hilar lymph nodes (0/1). After surgery, no specific treatment was initiated and at a follow-up visit 1 year after surgery, no local recurrence or metastasis was found.

**Conclusion:**

It is the largest cystic renal cell carcinoma that has ever been reported for laparoscopic resection. The selection of surgery for giant cystic renal cell carcinoma should be individualized. Retroperitoneal laparoscopy may be an option for such lesions.

## Background

As a malignant renal parenchymal tumor type, papillary renal cell carcinoma (PRCC) is the second most common form of renal cell carcinoma (RCC) and the most common non-clear cell RCC (1), accounting for approximately 15%-20% of all kidney cancers, and is associated with poor outcomes (2). More than 30% of RCC patients with localized disease will develop metastases after nephrectomy with an expected 5-year survival rate below 10% (3). Besides risk factors associated with RCC (smoking, hypertension, obesity, male, and family history), PRCC is uniquely related to renal dysfunction of different stages (4).

For the diagnosis of PRCC, typical PRCC radiographically appear as homogeneous, solid masses (5), while cystic lesions can be seen in around 25% of papillary tumors (6). Also, the characteristics of hypovascular in PRCC contributes to distinguish it from benign lesions on imaging examinations (7, 8). Based on computed tomography (CT) imaging criteria allowing for the analysis of renal cysts’ contour and contents, presence of septations and/or calcifications, and enhancement after contrast agent injection, the Bosniak classification system has been used to categorize renal lesions in an order of malignancy as follows: simple (I); minimally complicated (II); minimally complicated requiring follow-up (IIF); indeterminate (III); or cystic neoplasm (IV) (9). Complex renal cysts or solid components may be identified which requires more detailed characterization to determine the differential diagnosis and, thus, the appropriate treatment and prognostic evaluation. The low level of enhancement has led to tumors being misdiagnosed as renal cysts. As PRCC tumors are generally hypovascular, imaging with a single contrast phase displays lower enhancement compared to clear cell RCC (10). With multi-phase renal imaging, enhancement kinetics may provide additional discriminatory power. For instance, PRCC tumors tend to have peak enhancement in the later nephrographic phase of quadriphasic CT, whereas clear cell and chromophobe RCC show peak enhancement in the corticomedullary phase (11). Conventional imaging modalities, such as CT and MRI, have limitations, especially in terms of low sensitivity for early metastatic disease. Metastatic PRCC lesions may show similar enhancement characteristics to the primary tumor (5). However, dual-phase ^18^F-fluorodeoxyglucose (^18^F-FDG) positron emission tomography/computed tomography (PET/CT) showed better usefulness for predicting cell proliferation in RCC compared with single-phase imaging alone (12) and the potential to estimate the patient’s survival according to the accumulation measured maximum standardized uptake value (SUVmax) (13). A high SUVmax has demonstrated to correlate with disease aggressiveness in RCC and can improve detection of recurrent and metastatic RCC (14), and pretreatment max SUVmax assessed by FDG PET/CT was a useful prognostic marker for patients with advanced RCC (15).

Characterizing the molecular basis of PRCC and determining the main therapy goal is imperative for selecting the best strategy. Several mutated genes associated with PRCC have been identified including MET, NF2, SETD2, and Nrf2 pathway genes (16). Different molecular mechanisms are involved in PRCC biology. Mutations in the MET oncogene is present in the pathogenesis of hereditary PRCC forms found in a low rate of sporadic cases. Regarding the treatment of PRCC, localized sporadic PRCC can be managed with partial or radical nephrectomy, ablation, or active surveillance (17). There are few standard forms of treatment options available to patients with advanced PRCC, which has evolved to molecular targeted therapies and checkpoint inhibitors in the modern era. Several agents, including anti-VEGF drugs and mTOR inhibitors are possible options in treating advanced and metastatic PRCC (18), showing promising efficacy for PRCC (19, 20). In addition, MET inhibitors and checkpoint inhibitor therapy are highly anticipated based on the knowledge of hereditary papillary RCC and may be effective in advanced PRCC treatment (21).

In the study, we aimed to report a case of giant polycystic papillary renal cell carcinoma that underwent retroperitoneal laparoscopic radical nephrectomy by focusing on the diagnostic, clinical, and surgery to the patient. A 19-year-old male with polycystic lesions in the left kidney which was accidentally noted by enhanced CT (Bosniak IV, maximal diameter more than 18cm), underwent preoperative ^18^F-FDG PET-CT examination suggestive of malignancy with the SUVmax 4.8 of the lesion. The patient then underwent a retroperitoneal laparoscopic radical left nephrectomy successfully without cyst burst and no local recurrence or metastasis was found during the 1 year follow-up visit after surgery. Details follow.

## Case Presentation

**Figure 1:**
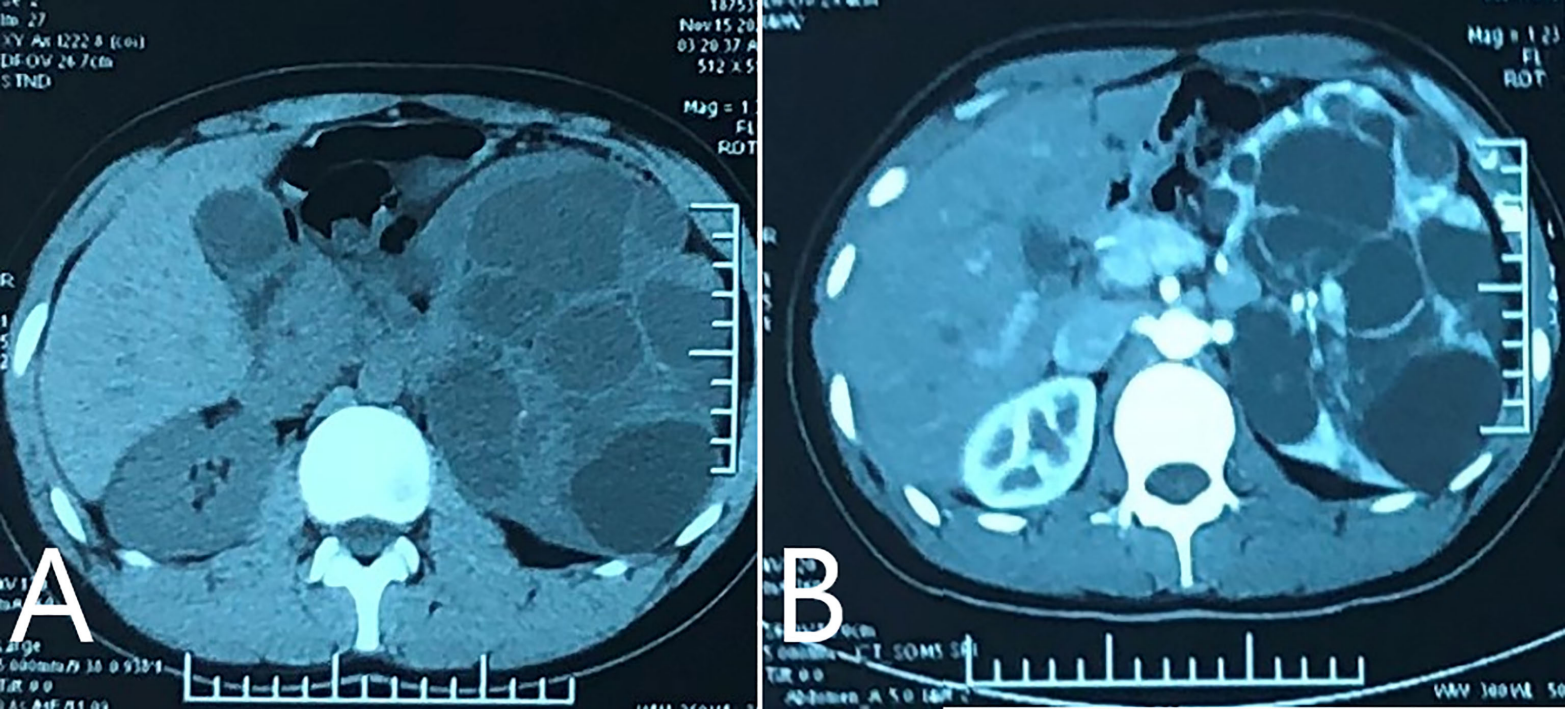
Enhanced CT performed on November 17^th^ 2020. **(A)** CT plain scan revealed multiple cystic lesions in left kidney, with solid component inside. The left kidney lost normal profile and enlarged significantly; **(B)** enhanced scan showed.

On December 7, 2020, a 19-year-old male was admitted to our hospital for polycystic lesions in the left kidney accidentally noted by computed tomography (CT). Three weeks prior, on November 17, 2020, enhanced CT, revealed solid component in multiple cystic lesions, which was enhanced after injection of contrast agent (**Figure 1**). According to Bosniak criteria (22), the lesion was classified into category IV.

The swollen kidney was palpable on the left abdomen and no other obvious abnormality was found in the physical examination. There was no previous personal or family medical history.

**Figure 2:**
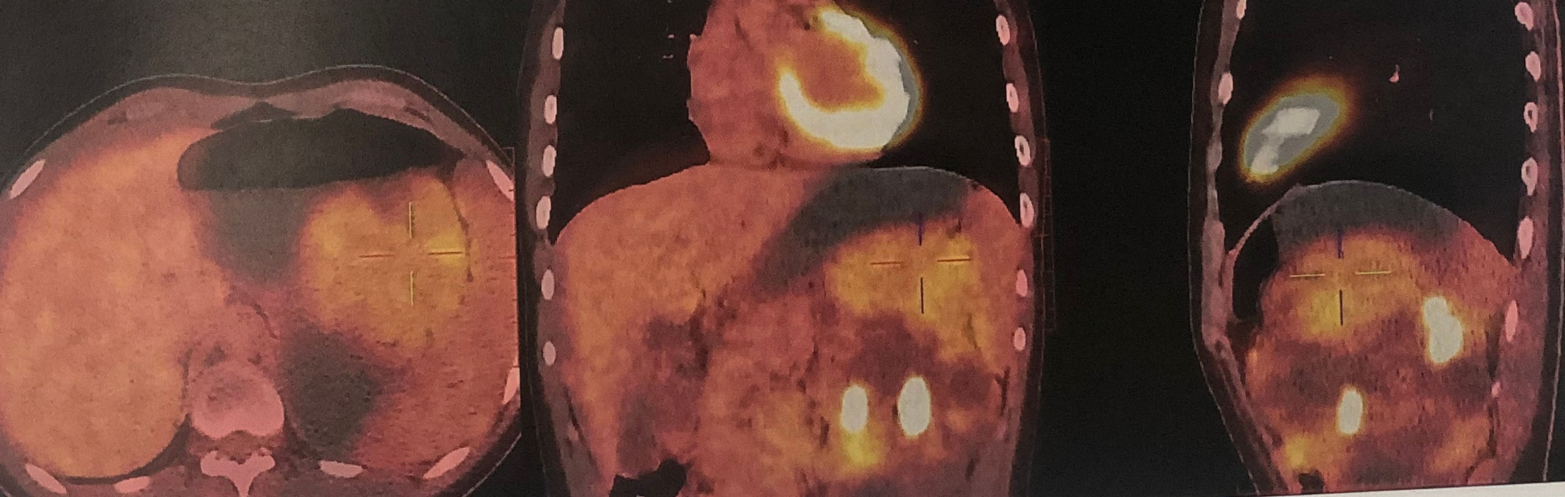
PET-CT found the left kidney was markedly enlarged, and there was a large cystic solid mass at the upper pole of the left kidney with uneven increased radiation uptake. The maximum cross section was about 13.8×10.9cm and the lesion SUVmax was 4.8, suggesting malignant disease. Multiple cysts of the left kidney were also seen.

After admission, positron emission tomography-computed tomography (PET-CT) using 18F-Fluorodeoxyglucose as the imaging agent (December 2, 2020) found the left kidney was significantly enlarged and a large cystic-solid mass was seen in the upper pole with uneven increase in radioactivity uptake. The maximum cross section was about 13.8×10.9cm and the lesion SUVmax was 4.8, suggesting malignant disease (**Figure 2**). No cysts or other abnormalities were observed in the contralateral kidney or other organs such as the liver. Imaging of renal blood flow showed only the upper part of the renal parenchyma was visible in the left kidney and the mass in the left kidney had a lack of blood supply. The kidney function was normal, with serum creatinine 71 µmol/L, blood urea nitrogen 3.58 mmol/L.

Given the young age of the patient, related gene tests were recommended to exclude genetic diseases or mutations but the patient refused to accept.

On December 10, 2020, laparoscopic radical resection of left kidney tumor was performed under general anesthesia without cyst rupture. During the surgery, the irregular enlargement of the left kidney was seen, the upper and lower diameter was about 15cm, and the transverse diameter was about 10cm. The left kidney and tumor were successful resected, with little intraoperative bleeding. One renal fossa drainage tube and one urinary tube were reserved during the operation. The removal of urinary tube was successful and the vital signs were stable after surgery.

Pathology: (left kidney) Combined with immunohistochemical, the lesions were consistent with papillary renal cell carcinoma (type 2 PRCC, WHO grade II), no renal capsule invasion, no renal pelvis and renal sinus fat involvement, no abnormality in ureter and renal arteriovenous end, no abnormality in a few adrenal tissues, chronic inflammation of hilar lymph nodes (0/1). Immunohistochemical results: PAX-8 (+), AE1/AE3 (+), CD10 (portion +), CK7 (-), EMA (+), P504 (portion +), CC (-), TFE3 (-), Vimentin (+), CA9 (-), CD117 (-). Next-generation sequencing of blood sample was performed after surgery, indicating no pathogenic/possibly pathogenic variation of TSC1/2 gene and other solid tumor susceptibility genes was detected.

The patient healed well and was discharged on December 15, 2020, without any complications. He received no further treatment after surgery. During a 1 year regular follow-up, the kidney function was normal, and no local recurrence or metastasis was found.

The patient signed informed consent for publication of this case report and accompanying images.

## Discussion

PRCC can be divided into two subtypes on the basis of histomorphologic features (23), namely, type 1 and type 2, with different clinical and genetic features (24, 25). Compared with type 1 PRCC, type 2 is more often associated with a greater stage, higher Fuhrman grade, higher frequency of necrosis and sarcomatoid features with worse outcome and more aggressive disease (18, 26). However, previous studies indicated that WHO/ISUP grade and tumor size were associated with the prognosis, rather than histologic subtype (24, 27). Based on preoperative imaging, this case was classified as Bosniak category IV and considered malignant. Further PET-CT examination also indicated a malignant lesion, which was proved by post-operative histological examination. Similar to these findings, despite pathologic findings of type 2 PRCC, there was no recurrence at 1 year of follow-up after surgical resection of the lesion due to its WHO grade 2 classification.

Surgery is the basic treatment of PRCC. Immunotherapy and cytotoxic chemotherapy are the treatment options other than surgery before the introducing of targeted therapy. A retrospective study that included 64 patients with metastatic non-clear-cell RCC histology analyzed the curative effect of immunotherapy and cytotoxic chemotherapy (28). The results showed metastatic non-clear-cell RCC patients are resistant to systemic therapy and poor survival, with the median overall survival time being 9.4 months (95% confidence interval, 8 to 14 months) (28). Type 2 PRCCs represent a heterogenous group with different genetic and molecular make up, making it difficult to apply effective targeted therapies (29). A previous study revealed that PRCC patients who underwent partial nephrectomy showed 5- and 10-year recurrence free survival of 95.8% and 73%, respectively (30). Given the increased recurrence rate more than 5 years after surgery, the patient still needs regular follow-up and imaging examinations for a longer time.

Giant cystic renal tumors are generally considered relatively contraindicated for laparoscopic surgery. The lesion size of this patient we present here was 18×17×17cm, with multiple cystic lesions. To our knowledge, this is the largest cystic renal cell carcinoma ever reported for successfully treated by laparoscopic surgery. The reasons for choosing laparoscopic surgery for this case are as follows: first, based on the patient’s age and imaging characteristics, the possibility of benign lesions cannot be ruled out, thus treatment with less invasive surgery is necessary and second, to explore the feasibility of retroperitoneal laparoscopy in the treatment of such huge polycystic kidney disease. Compared with open approach surgery, laparoscopic surgery can be performed under direct vision, the exposure of the operative area is more clear, and ligation and other surgical procedures are more reliable, ensuring the safety of surgery. Although the surgical procedure is relatively difficult, it indicates the possibility of successful retroperitoneal laparoscopic surgery for such lesions, providing an additional option.

## Conclusion

The selection of surgery for giant cystic renal cell carcinoma should be individualized. Retroperitoneal laparoscopy may be an option for such lesions.

## Data Availability Statement

The original contributions presented in the study are included in the article/supplementary material. Further inquiries can be directed to the corresponding author.

## Ethics Statement

Written informed consent was obtained from the participant for the publication of this case report.

## Author Contributions

ZH designed the study. ZH and HW collected the data and initially drafted the manuscript. ZJ review and revise the manuscript. All authors read and approved the final manuscript.

## Conflict of Interest

The authors declare that the research was conducted in the absence of any commercial or financial relationships that could be construed as a potential conflict of interest.

## Publisher’s Note

All claims expressed in this article are solely those of the authors and do not necessarily represent those of their affiliated organizations, or those of the publisher, the editors and the reviewers. Any product that may be evaluated in this article, or claim that may be made by its manufacturer, is not guaranteed or endorsed by the publisher.

## Abbreviations

CT, Computed tomography; PET-CT, Positron emission tomography-computed tomography; ^18^F-FDG, ^18^F-fluorodeoxyglucose; PRCC, Papillary renal cell carcinoma; RCC, Renal cell carcinoma; SUVmax, Maximum standardized uptake value.
